# Homeostasis of the Intraparenchymal-Blood Glutamate Concentration Gradient: Maintenance, Imbalance, and Regulation

**DOI:** 10.3389/fnmol.2017.00400

**Published:** 2017-12-05

**Authors:** Wei Bai, Yuan-Guo Zhou

**Affiliations:** Molecular Biology Center, State Key Laboratory of Trauma, Burn, and Combined Injury, Research Institute of Surgery and Daping Hospital, Third Military Medical University, Chongqing, China

**Keywords:** glutamate, blood–brain barrier, concentration gradient, brain diseases, glutamate transporter, endothelial cell

## Abstract

It is widely accepted that glutamate is the most important excitatory neurotransmitter in the central nervous system (CNS). However, there is also a large amount of glutamate in the blood. Generally, the concentration gradient of glutamate between intraparenchymal and blood environments is stable. However, this gradient is dramatically disrupted under a variety of pathological conditions, resulting in an amplifying cascade that causes a series of pathological reactions in the CNS and peripheral organs. This eventually seriously worsens a patient’s prognosis. These two “isolated” systems are rarely considered as a whole even though they mutually influence each other. In this review, we summarize what is currently known regarding the maintenance, imbalance and regulatory mechanisms that control the intraparenchymal-blood glutamate concentration gradient, discuss the interrelationships between these systems and further explore their significance in clinical practice.

## Introduction

Glutamate is the most important excitatory neurotransmitter in the central nervous system (CNS) (Zhou and Danbolt, 2014). It is synthesized and stored in specific glutamatergic neurons until released into the synaptic cleft in response to specific stimuli. It then acts on glutamate receptors (including ionotropic and metabotropic receptors) on pre- and post-synaptic membranes and astrocytes to mediate signal transduction. It thereby plays a broad range of important roles in the brain, including roles in neuronal development (Martin and Finsterwald, 2011), learning and memory (De Leonibus et al., 2003; Naie and Manahan-Vaughan, 2004), emotion (Swanson et al., 2005; Stan et al., 2014) and neuroinflammation (Dai et al., 2010). Once in the synaptic cleft, glutamate is either re-taken up by the presynaptic membrane or promptly removed by astrocytes that are wrapped around the synapse (Rose et al., 2009). However, if excess extracellular glutamate is not cleared in a timely manner, glutamate receptors on the post-synaptic membrane will be excessively activated, resulting in excitotoxic injury, including the destruction of the Ca^2+^ buffer system (Tymianski and Tator, 1996), free radical-induced damage to mitochondria (Perez Velazquez et al., 1997), and the inhibition of phosphatidylcholine-specific phospholipase C (PC-PLC) (Li et al., 1998). Abnormally high levels of cytosolic Ca^2+^ and the massive release of inflammatory mediators in turn trigger the exocytosis-like release of glutamate from synaptic terminals, which results in the extracellular accumulation of glutamate and an amplifying cascade of excitatory toxicity that finally leads to the dysfunction and degeneration of neuronal synaptic transmission (Sattler and Tymianski, 2001). The activation of ionotropic glutamate receptors can also produce neurotoxicity when uncoupled from neuroexcitation (Shen and Slaughter, 2002). Thus, the dramatic increase in intraparenchymal glutamate finally exacerbates the brain injury, leading to a poor prognosis (Schousboe and Waagepetersen, 2005).

Under normal conditions, blood glutamate levels are maintained in a steady state, and a normal diet prevents significant fluctuations in blood glutamate levels (Zlotnik et al., 2011a). In addition to the contributions of basic metabolic reactions, such as deamination and gluconeogenesis (Brosnan, 2000), it has more recently become clear that glutamate signaling has functions in non-neuronal tissues in sites as diverse as bone (Peet et al., 1999) and the pancreas (Morley et al., 2000), skin (Frati et al., 2000; Kinkelin et al., 2000) and lungs (Dai et al., 2013) because the same vesicular release and receptor-mediated responses that have been documented at synapses in the CNS have been observed in these tissues. Moreover, researchers have also found that an excitotoxic reaction is induced by high levels of blood glutamate in these tissues that is similar to that induced in the CNS (Skerry and Genever, 2001). In addition, our previous clinical results showed that high levels of blood glutamate are closely related to the occurrence of traumatic brain injury-induced acute lung injury (TBI-ALI) (Bai et al., 2017). These data further indicate that blood glutamate plays an important role in peripheral organs.

The intraparenchymal-blood glutamate concentration gradient is maintained in a relatively stable condition under physiological conditions (Hawkins and Vina, 2016). However, in a variety of brain diseases, the glutamate levels in the blood, cerebrospinal fluid (CSF) or both can significantly increase, and the normal intraparenchymal-blood glutamate concentration gradient is thereby disrupted (see **Table 1**). These events have serious consequences for the brain (Stefani et al., 2017; Yang S.J. et al., 2017) and peripheral tissues (Jang et al., 2004; Wen et al., 2015) and are associated with a worse prognosis (Egerton et al., 2014). Here, we reviewed what is currently known about how the intraparenchymal-blood glutamate concentration gradient is maintained and regulated and investigated the potential clinical significance and impact of changes in this gradient on various brain insults.

**Table 1:** Imbalanced intraparenchymal-blood glutamate concentration gradient in various brain insults.

Brain insults	Research in humans/animals	Intraparenchymal glutamate	Blood glutamate	Reference
**Acute brain injury**				
SAH	Humans/rats	↑		Nilsson et al., 1996; Bell et al., 2014
AIS	Humans/rats	↑	↑	Umemura et al., 1996; Castillo et al., 1997; Bonova et al., 2013
TBI	Humans/mice	↑	↑	Vespa et al., 1998; Li et al., 2008; Dai et al., 2013; Bai et al., 2017
ICH	Rabbits	↑		Qureshi et al., 2003
**Chronic disease**				
PD	Humans	↑**/**↓	N**/**↑	Iwasaki et al., 1992; Mally et al., 1997
AD	Humans	↑**/↓**	↑	Pomara et al., 1992; Miulli et al., 1993; Kuiper et al., 2000; Fayed et al., 2011
Epilepsy	Humans/mice	↑**/↓**		Smeland et al., 2013; Cavus et al., 2016
MS	Humans	↑	↑	Tisell et al., 2013; Azevedo et al., 2014; Al Gawwam and Sharquie, 2017
Schizophrenia	Humans	↓	↑	Song et al., 2014; Rowland et al., 2016


*AIS, acute ischaemic stroke; AD, Alzheimer’s disease; ICH, intracerebral hemorrhage; MS, multiple sclerosis; PD, Parkinson’s Disease; SAH, subarachnoid hemorrhage; TBI, traumatic brain injury.*

## The Formation and Maintenance of A Normal Intraparenchymal- Blood Glutamate Concentration Gradient

The glutamate concentration in the blood of healthy adults ranges from 40 to 60 μM (Bai et al., 2017). In some *in vitro* studies using acute brain slices, extracellular glutamate ranges from 25 to 90 nM (Cavelier and Attwell, 2005; Herman and Jahr, 2007; Le Meur et al., 2007); however, most *in vivo* studies using microdialysis, which is an FDA-approved method for clinical application, found much higher glutamate levels in brain, ranging from 0.2 μM to approximately 20 μM (Dash et al., 2009; De Bundel et al., 2011). Currently, researchers estimate a range from 1 to 10 μM in CSF or brain intercellular fluids (Hawkins, 2009; Li et al., 2009; Teichberg et al., 2009). Under normal conditions, the glutamate concentration is many times higher in the blood than in the CSF, and the difference between the two is nearly 50 μM, thus giving rise to the intraparenchymal-blood glutamate concentration gradient (Hawkins, 2009). The maintenance of intraparenchymal glutamate homeostasis is largely dependent on the integrity of the blood–brain barrier (BBB) limiting the influx of blood glutamate and the activity of endothelial glutamate transporters (EAATs), which constantly transport intraparenchymal glutamate into the blood (Cohen-Kashi-Malina et al., 2012).

### The Integrity of the BBB Is Required for a Normal Intraparenchymal-Blood Glutamate Concentration Gradient

Glutamate is prevented from moving between the intraparenchymal and blood compartments by the BBB with intact integrity. The BBB is a physical barrier that protects the CNS from invasion by toxic substances in the blood. It has a high electrical impedance (≈2000 Ω/cm^2^), and restricts even the passage of ions (Crone and Olesen, 1982; Sifat et al., 2017). The BBB is composed of brain microvascular endothelial cells and junctional complexes, an endothelial basement membrane and the astrocyte end feet that surround the endothelial cells. Each layer of the BBB plays a role in restricting the flow of solutes (Abbott, 2013; Tajes et al., 2014).

**FIGURE 1:**
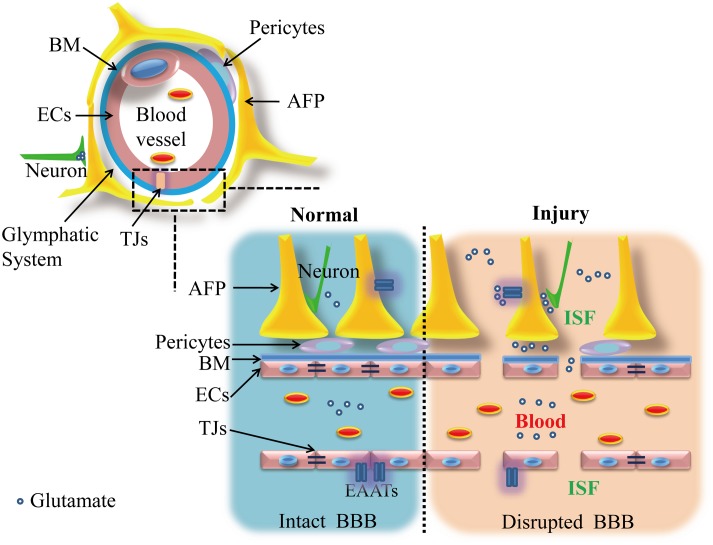
Illustration of the components of the blood–brain barrier (BBB) and distribution of glutamate under normal and injury conditions. Under normal conditions, the structure of the BBB is intact and includes a bilayer of endothelial cells (including TJs), astrocyte end feet and pericytes in combination with a basement membrane. These layers separate glutamate into two relatively isolated circumstances: brain and blood. However, after a brain injury, the BBB is disrupted, and the levels of glutamate in blood and brain both markedly increase. This figure was modified from Liu et al. (2016) with the permission of the authors. AFPs, astrocytic feet processes; BM, basement membrane; ECs, endothelial cells; EAATs, glutamate transporters; ISF, interstitial fluid; TJs, tight junctions.

Brain microvascular endothelial cells have more cytoplasmic vesicles and mitochondria than have been observed in the vessel endothelial cells of other tissues, in addition to more tight junctional complexes between cells (Oldendorf and Brown, 1975; Kniesel and Wolburg, 2000). These junctional complexes include adhesion junctions and tight junctions (TJs). The former are composed of cadherin–catenin and related proteins, while TJs mainly consist of three types of integral membrane proteins, including Claudins (Liebner et al., 2000a), Occludins (Furuse et al., 1998), and junctional adhesion molecules (JAMs) (Aurrand-Lions et al., 2001), in addition to a series of cytosolic accessory proteins, including members of the Zonula Occludens (ZO) family (Itoh et al., 1999; Wittchen et al., 1999) and cingulin (Sifat et al., 2017). These cytoplasmic proteins bind homotypically or assemble into heteropolymers, and they are responsible for the construction of the primary seal of TJs and essential for maintaining endothelial cell structure. In addition, the endothelial cell membrane is divided into the following two discrete parts by these TJs: the side facing the blood (called the luminal side) and the side facing the brain (called the abluminal side). Different populations of lipids and intrinsic proteins (e.g., glutamate transporters) reside in the luminal and abluminal spaces (Betz et al., 1980; van Meer and Simons, 1986; Tewes and Galla, 2001). The endothelial cells in the BBB are also surrounded by a continuous basement membrane that is mainly composed of collagen type IV, a variety of glycoproteins and pericytes. These proteins aggregate together to form a network that limits the flow of substances while simultaneously connecting with the surrounding tissue or extracellular matrix. They thereby play a supporting role in the BBB (Zhou et al., 2016). Embedded pericytes act alone and in association with endothelial cells or astrocytes to play key roles in maintaining the structural stability of the vessel wall (Siddharthan et al., 2007; Thanabalasundaram et al., 2010; Jo et al., 2013). Outside the basement membrane are enormous astrocyte end feet that surround approximately 85% of the surfaces of brain capillaries and play a role in regulating metabolism between brain vessels and neurons (Abbott et al., 2006). Thus, the basement membrane and astrocytic end feet are together considered the “second barrier” between the blood and brain (as shown in **Figure 1**).

**FIGURE 2:**
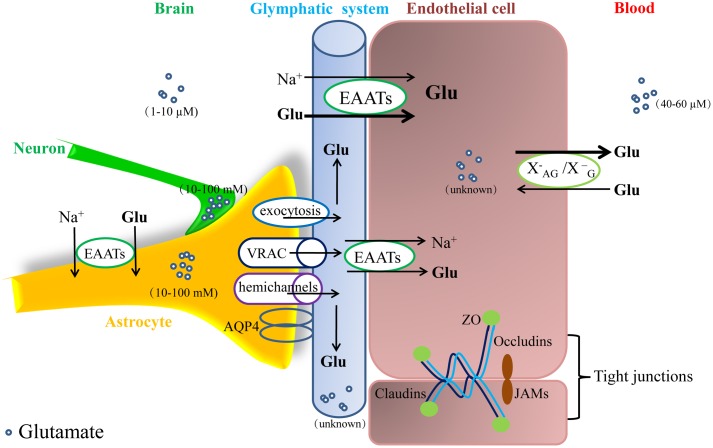
Glutamate metabolism and transport between the intraparenchymal and blood environments. The concentration of glutamate in the brain ranges from 1 to 10 μM, which is much lower than that in blood (40–60 μM) and astrocytes and neurons (10–100 mM). Under normal conditions, intraparenchymal glutamate is mainly dependent on EAATs on astrocytes and the abluminal membrane of endothelial cells for transport into cells. When the glutamate concentration in an endothelial cell exceeds the blood concentration, glutamate will be transported into the blood via facilitative transport ($X_{AG}^{-}/X_{G}^{-}$); however, it is difficult for blood glutamate to enter the brain via either tight junctions or carriers. EAATs, glutamate transporters.

Under physiological conditions, a high concentration of blood glutamate must cross at least five “films” (i.e., a bilayer of endothelial cells and astrocyte end feet in addition to the basement membrane) to enter the brain. In addition, a small amount of blood glutamate can be transported from the blood to the brain, and this process depends mainly on a Na^+^-independent carrier transporter (i.e., $X_{AG}^{-}/X_{G}^{-}$ transporters, which are mainly responsible for glutamate and aspartic acid) to be transported into endothelial cells, but this occurs at a low rate, and the carrier is close to saturation (Smith, 2000; Hawkins et al., 2006a; Hawkins, 2009); additionally, a non-saturation transport that relies on the pores between endothelial cells may allow a very low rate of blood-brain flux of glutamate (Al-Sarraf and Philip, 2003). Compared to other amino acids, glutamate is transported at a relatively low rate from the blood into the brain (Benrabh and Lefauconnier, 1996; Hawkins et al., 2006b). Thus, only a very small amount of blood glutamate can normally cross the BBB into the brain (Klin et al., 2010; Cederberg et al., 2014) (see in **Figure 2**).

### Na^+^-Dependent EAATs Are the Main Force Behind the Formation of a Normal Intraparenchymal-Blood Glutamate Concentration Gradient

Despite the presence of physical barriers, however, the isolation of each compartment is not complete, and there is mutual flow between them. An active brain-to-blood efflux against the concentration gradient is thought to be the principal mechanism underlying this exchange (O’Kane et al., 1999; Hawkins et al., 2006a; Hawkins and Vina, 2016), in which the Na^+^-dependent EAATs (hereafter referred to as EAATs) on endothelial cells are indispensable for the maintenance of the intraparenchymal-blood glutamate concentration gradient. EAATs are a family of high-homology transmembrane proteins that are composed of 500–600 amino acids and include EAAT_1_/GLAST, EAAT_2_/GLT-1, EAAT_3_/EAAC_1_, EAAT_4_, and EAAT_5_. They also share many similarities in their molecular structures, and they possess (van den Pol et al., 1990) 8 or 10 transmembrane segments, (Zhou and Danbolt, 2014) a serine-rich motif located within the cytoplasmic or extracellular loop of the cytoplasmic or transmembrane region that contains common functional domains related to substrate-binding, (Martin and Finsterwald, 2011) a glycosylation site in the second extracellular loop of each transporter, (De Leonibus et al., 2003) the same PKA/PKC phosphorylation-regulating sites, (Naie and Manahan-Vaughan, 2004) and a large hydrophobic region near the C-terminal that is different from that of other neurotransmitter transporters. These commonalities also determine their similar regulatory mechanisms (Kanai and Hediger, 1992; Kanai et al., 1995). The expression of EAATs varies between different tissues. GLT-1 and GLAST are mainly expressed in glial cells, neurons and endothelial cells in brain, alone or in concert. EAAC_1_ is prevalent in the CNS (including the retina) and is mainly expressed in post-synaptic neurons (Gegelashvili and Schousboe, 1997; Fontana, 2015). EAAT_4_ is highly enriched in Purkinje cells of the cerebellum (Massie et al., 2001), while EAAT_5_ is localized to two populations of glutamatergic neurons, bipolar neurons and photoreceptors in the retina (Lee et al., 2012). Thus, EAAT_1-3_ are the main transporters responsible for the vast majority of intraparenchymal glutamate transport in the brain. Many studies have demonstrated the necessity of EAAT_1-3_ in the maintenance and regulation of glutamate homeostasis under normal and pathological conditions (see in **Table 2**).

**Table 2:** Evidence for the necessity of EAAT_1-3_ in the maintenance and regulation of glutamate homeostasis.

Research in normal/disease states	Intervention	Effects	Reference
Normal	Delta(9)-THC	↓ GLAST/GLT-1, ↓ glutamate uptake	Castaldo et al., 2010
Normal	Ochratoxin A	↓ GLAST/GLT-1, ↓ glutamate uptake	Razafimanjato et al., 2010
Normal/hypoxic	BDNF/CoCl_2_	↑ GLAST, ↑ glutamate uptake	Dai et al., 2012
Alcohol consumption	Per2 mutant	↓ GLAST, ↑ intraparenchymal glutamate	Spanagel et al., 2005
Normal	Antisense oligonucleotide	↓ GLAST/GLT-1, ↑ intraparenchymal glutamate	Rothstein et al., 1996
Hearing loss	GLAST KO	↓ GLAST, ↑ intraparenchymal glutamate	Hakuba et al., 2000
Normal	Morphine	↓ EAAT3, ↑ extracellular glutamate	Guo et al., 2015


*BDNF, brain-derived neurotrophic factor; KO, knock out.*

Effective removal/uptake of excessive glutamate thus seems to be a crucial rescue mechanism, and failure or loss of the glutamate transport system may aggravate neurotoxic damage. In the CNS, a small proportion of the glutamate present in the synaptic cleft or intercellular fluid can undergo reuptake by the presynaptic membrane, but most of the extracellular glutamate is internalized into cells against a concentration gradient by EAATs located on glial cells or the endothelial cell membrane (Gegelashvili and Schousboe, 1998; Teichberg et al., 2009). One study has shown that when glutamate uptake is blocked, as little as 1 μM exogenous glutamate is sufficient to induce excitotoxic death in cortical neurons (Frandsen and Schousboe, 1990). Another study found that a 30-min exposure to 4 μM glutamate was sufficient to kill 50% of the neurons in astrocyte-poor cultures within 24 h, while 205 μM glutamate was required to kill the same percentage of neurons in astrocyte-rich cultures (Rosenberg et al., 1992). While the importance of the brain for blood efflux was confirmed by microinjection of radiolabelled glutamate and the kinetics of its appearance in blood (Hosoya et al., 1999; Gottlieb et al., 2003). In fact, glutamate uptake activity is so high that normal intact brain tissue is quite resistant to glutamate toxicity.

Endothelial glutamate transporters have a powerful scavenging ability that mainly depends on two processes: first and most important, EAATs are abundantly expressed on glial cells, especially astrocytes (Rothstein et al., 1994; Anderson and Swanson, 2000); additionally, astrocytes are rich in glutamine synthetase, which transforms extracellular glutamate into glutamine that can be pumped into cells to sustainably maintain a low concentration outside the cell (Danbolt, 2001). Thus, EAATs play a major role in the clearance of intraparenchymal glutamate (Zeng et al., 2010). Second, although the expression of EAATs is greatly reduced on endothelial cells (Lee et al., 2017), the brain is a highly vascularized organ [human brain contains approximately 100 million capillaries and a surface area of approximately 12 m^2^ (Bickel et al., 2001)]. In addition, almost every neuron in the brain has an adjacent capillary, and the average distance between a capillary and a neuron is only 8–20 μm (Schlageter et al., 1999). Therefore, EAAT-rich cerebral vessels and perivascular astrocyte end feet are particularly important for the formation and maintenance of intraparenchymal glutamate homeostasis. The driving force exerted by EAATs against the glutamate concentration gradient involves secondary active transport coupled to Na^+^, K^+^-ATPase (NKA). Intracellular Na^+^ is pumped out of the cell, while K^+^ is pumped into the cell, causing a Na^+^ concentration gradient from the extracellular to the intracellular compartment. EAATs cotransport one glutamate and three Na^+^ [or two Na^+^ and one H^+^ (Nicholls and Attwell, 1990)] into the cell. Assuming that the empty transporter is electrically neutral, the static charge of a full transport is one or two, and transport therefore generates electricity (Kanai et al., 1995).

Astrocytic and endothelial EAATs both play a crucial role in the regulation of intraparenchymal glutamate; however, endothelial EAATs play an important and unique function in the homeostasis of the intraparenchymal-blood glutamate concentration gradient since they lie directly between the brain and blood. A large number of studies have shown that endothelial EAATs are present only on the abluminal membrane (O’Kane et al., 1999; Helms et al., 2012). Under normal circumstances, the level of intraparenchymal glutamate depends on EAATs located on the endothelial abluminal membrane that transport it from the intraparenchymal space to the blood. At present, a widely accepted and confirmed view is that extracellular glutamate in the brain is continuously transported into microvascular endothelial cells, which become enriched, by abluminal EAATs against a glutamate concentration gradient. When the glutamate concentration in an endothelial cell exceeds the blood concentration, glutamate will be transported into the blood via facilitative transport (O’Kane et al., 1999; Hawkins et al., 2006a). This process is also called “concentration climbing” and is considered the most important mechanism for forming and maintaining the intraparenchymal-blood glutamate concentration gradient under physiological conditions (Helms et al., 2012). Previously, one plausible mechanism was proposed that glutamate efflux from brain extracellular fluids into the blood might involve a “glutamine-glutamate cycle” (Lee et al., 1998; O’Kane et al., 1999). In such a mechanism, the uptake of excess glutamate into astrocytes leads to its conversion into glutamine, which is released by astrocyte end feet and is subsequently pumped into endothelial cells via glutamine transporters and then converted back into glutamate. However, the results of further experiments using an isolated BBB model quickly eliminated the hypothesis that a “glutamate–glutamine” cycle makes no contribution to brain endothelial cell uptake of intercellular glutamate (Cohen-Kashi-Malina et al., 2012). In addition to this process, the glutamate in intercellular fluids can first be taken up into astrocyte end feet and then excreted via exocytosis (Wilhelm et al., 2004; Crippa et al., 2006) and volume-regulated anion channels (VRAC) (Takano et al., 2005) and hemichannels (Ye et al., 2003) before being transported into endothelial cells by glutamate transporters (as shown in **Figure 2**).

### The Contribution of the Glymphatic System to the Formation of a Normal Intraparenchymal-Blood Glutamate Concentration Gradient

A long-held anatomical view states that the brain lacks a lymphatic system but instead uses CSF reflux. However, recent studies have suggested that in addition to endothelial cells, a novel pathway operates at the blood–brain interface, which may involve a separate paravascular highway that facilitates the rapid exchange of CSF and tissue fluids. This pathway has been referred to as the “glymphatic system” (Iliff and Nedergaard, 2013). The glymphatic system (or glymphatic clearance pathway) is a functional waste clearance pathway in the vertebrate CNS. The pathway consists of a para-arterial influx route by which CSF enters the brain parenchyma coupled to a clearance mechanism by which interstitial fluid (ISF) and extracellular solutes are removed from the interstitial compartments of the brain and spinal cord. The exchange of solutes between the CSF and the ISF is driven by arterial pulsation (Iliff et al., 2013) and regulated during sleep by the expansion and contraction of the brain extracellular space (Mendelsohn and Larrick, 2013). The clearance of soluble proteins [such as beta amyloid (Abeta), phosphorylated tau (p-tau) and Apolipoprotein E (apoE)] (Iliff et al., 2012; Achariyar et al., 2016; Sullan et al., 2017), waste products (such as lactate) (Lundgaard et al., 2017), and excess extracellular fluid (which contain small-molecule intraparenchymal glutamate and other brain injury markers) (Yang et al., 2013; Thrane et al., 2014; Plog et al., 2015) is accomplished via the convective bulk flow of the ISF and facilitated by astrocytic aquaporin 4 (AQP4) water channels (see in **Figures 1**, **2**).

## The Intraparenchymal-Blood Glutamate Concentration Gradient Is Imbalanced Under Pathological Conditions

Despite controversy, most studies have found that when various types of acute brain injury, such as subarachnoid hemorrhage (SAH), acute ischaemic stroke (AIS), intracerebral hemorrhage (ICH), or TBI occurs, glutamate levels in the brain and blood can reach extremely high levels. This elevation has also been observed in patients with chronic brain diseases, such as Parkinson’s Disease (PD), Alzheimer’s disease (AD), epilepsy, multiple sclerosis (MS) and schizophrenia (see in **Table 1**). Glutamate levels in the blood and CSF are significantly higher in patients with these conditions than in normal individuals, and the intraparenchymal-blood glutamate concentration gradient is also dramatically increased.

### Sources of Elevated Intraparenchymal Glutamate

A tremendous amount of glutamate is stored in brain neurons and glial cells at a concentration of up to 10–100 mM (Ma et al., 2012; Mehta et al., 2013). Previous studies have shown that when a brain injury occurs, in addition to the direct destruction of neurons and glial cells, a massive amount of glutamate is released into the brain intercellular fluid by other mechanisms (Katayama et al., 1990). These include external Ca^2+^- or intracellular Ca^2+^-dependent vesicular release (Drejer et al., 1985; Katchman and Hershkowitz, 1993), release via swelling-activated anion channels (Bednar et al., 1992), an indomethacin-sensitive process in astrocytes (Parpura et al., 1994; Hassinger et al., 1995; Bezzi et al., 1998), and glutamate transporter dysfunction (Szatkowski et al., 1990; Szatkowski and Attwell, 1994). By using blockers that affect each release mechanism, researchers have demonstrated that glutamate release is largely caused by the dysfunction of glutamate transporters (Rossi et al., 2000). Conversely, this is due in part to a reduction in the expression of these transporters. For example, in patients with TBI, researchers found that reduced survival and degeneration in astrocytes resulted in a significant decrease in the expression of EAAT_1/2_ within 7 days after injury (van Landeghem et al., 2006; Beschorner et al., 2007), and shear or inertial force also caused changes in EAAT expression and activity-associated astrocyte deformation in TBI (Unger et al., 2012). In contrast, in such cases, the dysfunction of EAATs manifested as decreased activity and reduced transport efficiency, but reverse transport remained possible. In acute ischaemia in the hippocampus, reversed transport of neuronal EAATs resulted in a sharp increase in extracellular glutamate levels (Rossi et al., 2000). Moreover, under inflammatory conditions, which consistently accompany brain insults, the release of pro-inflammatory cytokines not only inhibited glutamate scavenging capacity (Fine et al., 1996) but also reduced EAAT_2_ expression in astrocytes (Rozyczka et al., 2004; Sitcheran et al., 2005).

Previously, there has been widespread controversy regarding whether the BBB is severely physically destroyed following brain injury. Although some reports have suggested that the structure of the BBB after brain injury becomes damaged and loses part of its barrier function (van Vliet et al., 2007; Weissberg et al., 2014), most studies nevertheless suggest that the direct damage (i.e., vascular rupture caused by brain contusion) is limited (Zhao et al., 2015), but that its functional components (i.e., junctional complexes between endothelial cells) might suffer more severe damage (Zhao et al., 2015; Price et al., 2016), which could last for a long time (Jiao et al., 2011). Under pathological conditions, such as shock or inflammation or the presence of a tumor, the expression of Claudin-1 and Occludin dramatically decrease in blood vessels (Liebner et al., 2000b; Papadopoulos et al., 2001). Experiments in which dye was injected into animals (Abdul-Muneer et al., 2013; Hue et al., 2014) or magnetic resonance imaging (MRI) in humans (Merali et al., 2017) confirmed the increased permeability of the BBB after a brain injury. The results of these studies suggest that increased blood glutamate can also penetrate into the brain via the functionally impaired BBB and play specific roles in the increased intraparenchymal glutamate observed after brain injury (shown in **Figure 1**).

The transport of intraparenchymal glutamate by the glymphatic system can also be decreased. In a variety of animal models of brain insult, including models of AD (Yang J. et al., 2017), aging (Kress et al., 2014), epilepsy (Hubbard et al., 2016), and ICH (Qiu et al., 2015), AQP4 expression is decreased and its polarity is impaired. The outflow of CSF was significantly lower in AQP4 knockout (KO) mice than in wild type mice, and the clearance rate of intercellular fluid was also greatly reduced (Plog et al., 2015). However, while the decrease in its expression had largely normalized by 7 days post-injury, AQP4 depolarization continued to be observed (Zhao et al., 2017). Moreover, a separate study also found that AQP4 is co-expressed with GLT-1 on brain perivascular astrocytes, whereas genetically knocking out AQP4 inhibited the expression of GLAST, resulting in the inhibition of intraparenchymal glutamate efflux (Li et al., 2014).

### Sources of Elevated Blood Glutamate

The sources of the observed elevation in blood glutamate levels have remained unclear. Researchers have analyzed the rate of glutamate uptake in various peripheral tissues and organs after intravascular injection of [^14^C]-Glu and found that skeletal muscle contain the body’s largest storage pool of glutamate, accounting for approximately 59% of the total storage amount (Klin et al., 2010). In patients with acute spinal cord injury (SCI), researchers have found that an ion distribution disorder caused by the abnormal expression of NKA and its FXYD1 subunit in skeletal muscle may be the molecular basis underlying the release of glutamate from skeletal muscle after injury (Boon et al., 2012). Blood cells are another important source of blood glutamate. A comparison of patients with cerebral infarction and healthy controls has revealed that the glutamate-releasing ability of platelets is reduced in patients, suggesting that at the onset of a brain infarct, platelets are activated, which frees up a large amount of glutamate to enter the blood (Aliprandi et al., 2005; Morrell et al., 2008). Additionally, *in vitro* experiments have shown that the endothelial barrier function is altered by the release of soluble polymorphonuclear leukocyte (PMN)-derived glutamate during inflammatory states (Collard et al., 2002). In addition, bone might be another source of glutamate because osteoclasts also secrete L-glutamate in a Ca^2+^-dependent manner when stimulated with KCl or ATP (Morimoto et al., 2006).

## The Regulatory Mechanisms Underlying the Imbalance in the Glutamate Concentration Gradient

The key processes goals when resolving imbalances in intraparenchymal-blood glutamate homeostasis are to reduce the elevated glutamate levels in both the blood and the brain, which includes preventing the entry of blood glutamate into the brain and enhancing the transport efficiency of glial and endothelial EAATs and the glymphatic system under pathological conditions. Thus, based on the previously discussed mechanism underlying the formation of the intraparenchymal-blood glutamate concentration gradient, we now review the regulatory mechanisms involved in modulating EAATs, TJs, the glymphatic system and glutamate itself.

### Regulatory Mechanisms That Affect EAATs Expression and Function

Many factors were involved in the mRNA or protein turnover of EAATs to regulate their expression and distribution. Both glutamate and kainite dramatically increase GLAST protein expression in cultured astrocytes without significantly increasing the amount of GLAST mRNA (Gegelashvili et al., 1996). L-DOPA and ceftriaxone both increase GLT-1 expression, but they exert opposite effects on the intraparenchymal glutamate (Robelet et al., 2004; Lee S.G. et al., 2008). Studies have suggested that proteins translated from aberrant mRNAs may undergo rapid degradation and/or produce a dominant-negative effect on normal EAAT_2_ proteins that reduces the amount of the protein and its activity (Lin et al., 1998). Hence, while a glutamate transporter and the expression of its corresponding mRNA can differ according to the cell phenotype, cellular environment and locally active signaling pathways, the specific mechanism underlying these differences is unknown (Gegelashvili and Schousboe, 1997). Studies examining these differences at the post-translational level have primarily focused on modifications of EAATs, including their phosphorylation, glycosylation, and ubiquitination. Previous experiments have confirmed that the expression and uptake efficiency of EAATs are dependent on a PKA/PKC pathway (Casado et al., 1993; Figiel and Engele, 2000; Huang et al., 2006), and amelioration of the delayed ischaemic brain damage can be achieved by increasing both the expression and function of EAAT_1_ via these pathways (Yanagisawa et al., 2015; Karki et al., 2017). The effect of glycosylation on EAATs remains controversial. Observations of the *N*-glycosylation of GLAST demonstrated that the kinetic characteristics of GLAST are not affected (Conradt et al., 1995), while another study of EAAC_1_ found that glycosylation may be necessary for the activity of the transporter under hypertonic stress (Ferrer-Martinez et al., 1995). However, no clear mechanism has been identified to explain how this glycosylation is regulated. Recent evidence indicates that the turnover of EAATs in the plasma membrane is accelerated by an ubiquitin-dependent process, which is triggered by the activation of PKC (Gonzalez-Gonzalez et al., 2008; Garcia-Tardon et al., 2012). Amphetamine triggers the internalization of EAAT_3_ but simultaneously produces a dose-related increase in extracellular concentrations of glutamate (Del Arco et al., 1999; Underhill et al., 2014) (see also **Table 3**).

**Table 3:** Factors involved in the regulation of EAATs and TJs.

Factors involved	*In vivo*/*In vitro* studies	Effects	Reference	
**EAATs**				
Glutamate and kainite	*In vitro*	↑ GLAST protein expression without mRNA change	Gegelashvili et al., 1996	
L-DOPA	*In vivo*	↑ GLT-1expression	Robelet et al., 2004	
Ceftriaxone	*In vitro*	↑ EAAT_2_ expression	Lee S.G. et al., 2008	
Arundic acid	*In vivo*/*In vitro*	↑ EAAT_1_ expression ↑ EAAT_1_ activity (phosphorylation)	Yanagisawa et al., 2015; Karki et al., 2017	
Hypertonic stress	*In vitro*	↑ EAAC_1_ activity (glycosylation)	Ferrer-Martinez et al., 1995	
Amphetamine	*In vitro*	↑ EAAT_3_ endocytosis	Underhill et al., 2014	
**TJs**				
Ca^2+^	*In vitro*	↑ migration of ZO-1	Stevenson and Begg, 1994
Insulin	*In vivo*/*In vitro*	↑ TJs integrity	Sun et al., 2015; Ito et al., 2017
Dexmedetomidine	*In vivo*/*In vitro*	↑ ZO-1 and Occludin expression	Hue et al., 2015; Liu et al., 2015
IFN-γ	*In vivo*/*In vitro*	↓ TJs protein expression	Chai et al., 2014; Haroon et al., 2014
Glutamate	*In vivo*	↑ BBB permeability	Vazana et al., 2016
A_2A_R	*In vivo*	↓ TJs protein expression	Li et al., 2009; Carman et al., 2011


*A_*2A*_R, adenosine 2A receptor; EAATs, glutamate transporters; L-DOPA, 3-(3,4-Dihydroxyphenyl)-L-alanine; TJs, tight junctions.*

Because glutamate transport is associated with ion transport, the regulation of ions may significantly impact the function of transporters. In shock or TBI cases, ischaemia and hypoxia lead to a deficiency in energy synthesis, and ATP deficiency-induced mitochondrial dysfunction directly affects Na^+^-K^+^ pumps and Na^+^-Ca^2+^ and Na^+^-H^+^ exchange, resulting in a disordered charge distribution both in and outside the cell membrane. The transfer efficiency is then decreased, and in some cases, uncontrolled reverse transport occurs, resulting in the release of glutamate. Hence, these processes can eventually result in high concentrations of extracellular glutamate (Szatkowski et al., 1990; Rossi et al., 2000; Zhang et al., 2007). When the intracellular Na^+^ concentration increases from 15 to 30 mM, glutamate transporters began to reverse transport. However, when a Na^+^-H^+^ antiporter inhibitor was applied, it induced rapid extensive intracellular acidosis and glutamate transporter reversal but not an overload of intracellular Na^+^. H^+^ may therefore play an equally important role in regulating the direction in which EAAT is transported with Na^+^ (Gemba et al., 1994; Longuemare et al., 1999). Although the number of studies examining glutamate transporters has gradually increased, the precise mechanisms by which Na^+^, K^+^, and H^+^ lead to reverse transport remain unresolved and require further investigation (Machtens et al., 2015). In addition to these mechanisms, there may also be other routes by which extracellular glutamate concentrations can be quickly altered, including the regulation of EAAT activity, and this topic is worthy of further exploration.

### Pathways Involved in the Regulation of Endothelial TJ Expression

Many signaling pathways and endo/exogenous factors have been shown to regulate the assembly of TJs (Izumi et al., 1998). Abnormalities in Ca^2+^ homeostasis have been implicated in the pathophysiology of brain injury (Sun et al., 2008), and one reason for this phenomenon is that Ca^2+^ is tightly connected to the regulation of TJs both in and outside the cell (Lacaz-Vieira and Marques, 2003). Changes in intracellular Ca^2+^ levels can trigger a series of PKA- or PKC-mediated molecular events that increase transendothelial resistance and promote the migration of ZO-1 from the cytoplasm to the membrane (Stevenson and Begg, 1994). Brain injury is consistently accompanied by alterations in hormones, such as insulin (Jing et al., 2017) and cortisol (Alain-Pascal et al., 2010), which also play an important role in the regulation of TJs. In both an *in vitro* model of BBB and *in vivo* research, insulin and dexamethasone were found to rapidly increase the expression of TJs and decrease permeability (Hue et al., 2015; Liu et al., 2015; Sun et al., 2015; Ito et al., 2017). Researchers observed a significant decrease in blood glutamate after injection of insulin (Zlotnik et al., 2011b); however, there was no significant correlation between blood glutamate levels and brain uptake of glutamate (Hawkins et al., 2010); in contrast, dexamethasone greatly augmented the intraparenchymal glutamate level after ischaemia (Chen et al., 1998). Blocking some chemokines/cytokines, such as IFN-γ, ameliorates both the disruption of BBB permeability and the down-regulation of TJ protein expression (Chai et al., 2014), and an increase in cortex glutamate has been observed after treatment with IFN-γ (Haroon et al., 2014). Intraparenchymal glutamate itself also induces changes in BBB permeability (Vazana et al., 2016). Additionally, the rapidly increased adenosine acting on the adenosine 2A receptor (A_2A_R) after a brain injury can cause cytoskeletal changes in endothelial cells while simultaneously reducing the expression of TJs, thereby increasing BBB permeability (Chen et al., 2007; Carman et al., 2011). Furthermore, the elevated intraparenchymal glutamate level is counteracted by A_2A_R inactivation (Li et al., 2009) (see also **Table 3**).

### The Function of the Glymphatic System Is Closely Related to AQP4

The results of several studies have confirmed that astrocytic AQP4 plays an importantly role in clearance in the glymphatic system (Iliff et al., 2013, 2014; Murlidharan et al., 2016). As previously stated, anatomical associations have supported the notion of an interaction between endothelial cells and astrocytes (Abbott et al., 2006). Research has shown that endothelial cells promote the accumulation of AQP4 by exerting an inductive effect on extracellular matrix components such as agrin and via direct mechanical interactions with end foot processes (Camassa et al., 2015). Using an *in vitro* model of BBB, the results of another study confirmed that both the amount and localization of AQP4 protein in astrocytes were influenced by direct contact with endothelial cells (Haruki et al., 2013). In addition, the expression of AQP4 is influenced by other factors. Progesterone significantly reduced AQP4 expression in peri-contusion areas (Guo et al., 2006), the activation of P2X7R in astrocytes was associated with the down-regulation of AQP4 in rat brain astrocytes (Lee M. et al., 2008), and in our previous experiments, we found that AQP4 expression was significantly lower in the brain cortex in A_2A_R KO mice than in wild type controls following brain blast injury, suggesting that A_2A_R activity may affect the expression of AQP4 (Ning et al., 2013).

### Pathways and Regulation of Glutamate Itself

Many enzymes or substrates are directly involved in the metabolic process of glutamate. Glutamate dehydrogenase (GDH) is important in the transdeamination of glutamate, as activation of GDH not only significantly decreases the glutamate concentration in brain (Lee et al., 2005) but also restores alpha-ketoglutarate (alpha-KG) and ATP levels after brain ischaemia (Kim et al., 2017) and increases glutamate uptake in the forebrain (Whitelaw and Robinson, 2013). Glutamine synthetase (GS) plays a key role in intraparenchymal glutamate metabolism, as after ischaemia, an increase in GS in astrocytes occurs rapidly and in parallel with proliferative changes in astrocyte organelles (Petito et al., 1992). In blood, the substrates of glutamate oxaloacetate transaminase (GOT) and glutamate pyruvate transaminase (GPT), oxaloacetate and pyruvate have also demonstrated powerful scavenging capacity (Zlotnik et al., 2012).

## The Interrelationship Between Intraparenchymal and Blood Glutamate

As previously mentioned, glutamate does not exist in isolation in the brain or blood. In one study, as the glutamate concentration rose from 1 to 500 μM in the carotid artery in primary hypertension rats, the rate at which glutamate penetrated the brain increased (Al-Sarraf and Philip, 2003); additionally, systemic injection of glutamate has been reported to aggravate brain damage (Zlotnik et al., 2012). Another study showed that intravenous administration of aspartate aminotransferase (AST) (Ruban et al., 2012), pyruvate and oxaloacetate (Zlotnik et al., 2009, 2012) could significantly reduce glutamate levels in the blood in addition to accelerating the discharge of glutamate from the brain, decreasing intraparenchymal glutamate levels (Teichberg et al., 2009), significantly improving prognoses and outcomes (Campos et al., 2011), and extending the lifetimes of the mice (Zlotnik et al., 2007, 2009; Klin et al., 2010). These findings indicate that the environments in the brain and blood are mutually influenced, and blood glutamate is of great significance for the brain. However, the effect of elevated blood glutamate on the concentration of intraparenchymal glutamate and whether it is also an important source of the rapid increase in intraparenchymal glutamate remain poorly understood. Moreover, while there is no direct evidence showing that intraparenchymal glutamate levels influence blood glutamate levels, the results of our recent studies in patients with TBI indicate that the severity of brain injury is positively associated with blood glutamate levels (Bai et al., 2017).

## The Significance of Potential Applications That Alter the Homeostasis of the Intraparenchymal-Blood Glutamate Concentration Gradient

In an effort to ensure that “CNS security” is made an appropriate priority in pathological cases, administering a glutamate receptor antagonist following a brain insult has, in many pre-clinical studies, indicated neuro-protective roles and improved prognoses (Furukawa et al., 2003; de Miranda et al., 2016). However, the results of clinical trials have suggested that these drugs fail to improve long-term prognoses or reduce mortality after brain injury (Davis et al., 1997; Lees, 1997; Maas et al., 2006). We hypothesized that this might be because the important role of blood glutamate (and therefore the intraparenchymal-blood glutamate concentration gradient) was ignored. Because haemofiltration (Rogachev et al., 2012) and peritoneal dialysis (Rogachev et al., 2013) have been approved to efficiently lower blood glutamate levels in patients, these measures could be used to treat acute and chronic brain disorders that are accompanied by elevated glutamate levels in both the brain and blood.

However, many unsolved issues remain. For example, is peripheral glutamate an important source of increased intraparenchymal glutamate following a brain injury? Can blood glutamate act as a diagnostic or prognostic indicator of brain injury? Only by increasing our understanding of the generation and metabolism of intraparenchymal-blood glutamate can we identify methods to regulate the glutamate concentration gradient at the source and thereby prevent the damaging effects of high levels of glutamate. Such studies would offer important and effective methods for treating the acute phase of brain injury.

## Author Contributions

Both the authors, WB and Y-GZ, listed have made a substantial, direct and intellectual contribution to the work, and approved it for publication.

## Conflict of Interest Statement

The authors declare that the research was conducted in the absence of any commercial or financial relationships that could be construed as a potential conflict of interest.
